# Vermont

**Published:** 1896-12

**Authors:** 


*Vermont.* Governor Grout received among the first bills sent him one relative to tuberculosis. It provides that the State Board of Health be instructed to examine the condition of ventilation and sanitation of stables in which cattle are kept, to provide for a series of experiments to be conducted at the experiment station at Burlington, Vt., investigating the possibility of recovery of infected cattle, results of such investigations to be published. After December 1, 1897, no indemnity to be paid for cattle condemned unless it is ascertained that such stables had been previously placed in condition to resist infection and dissemination.

